# Predictive factors of spontaneously regressed uterine endometrial polyps during the waiting period before hysteroscopic polypectomy

**DOI:** 10.1186/s13256-021-02982-4

**Published:** 2021-08-01

**Authors:** Asuka Okamura, Eriko Yano, Wataru Isono, Akira Tsuchiya, Michiko Honda, Ako Saito, Hiroko Tsuchiya, Reiko Matsuyama, Akihisa Fujimoto, Osamu Nishii

**Affiliations:** Department of Obstetrics and Gynaecology, University Hospital Mizonokuchi, Teikyo University School of Medicine, Kanagawa, 5-1-1 Kawasaki, Takatsu-ku, Futago, Kanagawa 213-8507 Japan

**Keywords:** Hysteroscopic polypectomy, Multivariate analysis, Retrospective study, Uterine endometrial polyp, Spontaneous regression

## Abstract

**Background:**

The presence of uterine endometrial polyps is associated with not only abnormal uterine bleeding but also infertility, so the use of hysteroscopic polypectomy has been increasing. This operation is considered to increase cost-effectiveness when performed prior to infertility treatments. However, there are typical problems to consider, including the possibility of spontaneous regression of the polyp and the duration of complete endometrial wound healing after surgery. Meaningless interventions must be avoided, when possible. Therefore, data acquisition and analysis of various findings obtained from surgery have become important for improving treatment procedures and patient selection. To estimate the spontaneous regression rates and contributions of multiple factors to uterine endometrial polyps during the waiting period (approximately 2–3 months) before hysteroscopic polypectomy, we performed a multivariate analysis of data from the records in our hospital.

**Methods:**

The medical records of 450 cases from September 2014 to April 2021 in our hospital were retrospectively reviewed under the approval of our Institutional Review Board. We included all cases of hysteroscopic polypectomy with postoperative pathological diagnosis. We defined cases as having a “spontaneously regressed polyp” when the target polyp was not detected by postoperative pathological examination. We extracted data on the following ten factors: “Advanced age” (≥ 42 years), “Small polyp” (< 10 mm), “High body mass index” (≥ 25 kg/m^2^), “Nulliparity,” “Single polyp,” “Infertility,” “Hypermenorrhea,” “Abnormal bleeding,” “No symptom,” and “Hormonal drug use.” We also classified cases into five groups according to the size of the polyp (≤ 4.9 mm, 5.0–9.9 mm, 10.0–14.9 mm, 15.0–19.9 mm, and ≥ 20.0 mm) and determined the frequency of spontaneously regressed polyp in each group.

**Results:**

After exclusion of cases with insufficient data or other diseases, such as submucosal leiomyoma, 424 cases were analyzed. Among them, 28 spontaneously regressed polyps were identified, and the highest frequency of spontaneously regressed polyp was detected among the cases with polyps measuring 5.0–9.9 mm (16.4%). On multivariate analysis of the ten factors, “Small polyp” and “Hormonal drug use” were found to significantly impact the frequency of spontaneously regressed polyp.

**Conclusions:**

On the basis of the factors identified in this analysis, the indications for observation or medical therapy adapted to small polyps might be expanded.

## Background

Uterine endometrial polyps are common gynecological diseases and are associated with clinical symptoms, such as abnormal bleeding and infertility [1]. Symptomatic polyps, which cause abnormal bleeding and hypermenorrhea, are found by means of direct investigation during either an outpatient hysteroscopy or transvaginal ultrasonography, whereas asymptomatic polyps are incidentally discovered during a workup aimed to determine the cause of infertility. According to a previous study, endometrial polyps have been detected in up to 25% of women with unexplained infertility [2, 3]; however, no guidelines exist for monitoring these types of patients. Some studies have concluded that surgical resection performed prior to the application of infertility treatments is generally successful [4, 5]. Therefore, hysteroscopic polypectomy has been performed with increasing frequency. However, such examinations are not believed to have an impact on infertility treatments when the discovered polyp is small [6] since small polyps frequently regress spontaneously within 1 year [1, 7, 8]. Moreover, in our hospital, there have been cases in which target polyps could not be detected during operations, despite being detected by outpatient hysteroscopic examination. Although pregnancy outcomes are not compromised by hysteroscopic polypectomy performed before infertility treatments, most clinicians recommend a 1- to 2-month period between the two procedures to allow for endometrial wound healing [9]. These recovery periods are lengthy, particularly for infertile women of an advanced age. Moreover, a 1-year follow-up that confirms small polyp regression is impractical and burdensome for these infertile patients.

Thus, we retrospectively reviewed the medical records of patients who underwent hysteroscopic polypectomy and extracted data for cases of “spontaneously regressed polyp” (SRP) during the waiting period for surgery. That is, we compiled the detailed characteristics of cases with spontaneous polyp regression that occurred in a relatively short period. We then collected data on the size of the target polyp at the time of the diagnostic visit and outpatient hysteroscopic examination. The results of the pathological examination after surgery were also investigated. In doing so, we identified the clinical characteristics that influence polyp regression and determined the indications for hysteroscopic polypectomy.

## Methods

### Data collection

This study was reviewed and approved by the Human Ethical Committee of the University of Teikyo Hospital (Trial registration number: 17-193). The deidentified medical records of 450 female patients who underwent hysteroscopic polypectomy from 1 September 2014 to 30 April 2021, were reviewed retrospectively. Uterine endometrial polyps were diagnosed as smooth-margined masses in the endometrium by transvaginal ultrasound and office hysteroscopy. The maximum length of the target polyp was measured by transvaginal ultrasonography before hospitalization (that is, when the original diagnosis was made) in an outpatient setting. In most cases, the size was measured by injecting water into the uterine cavity during office hysteroscopic examination. We excluded 23 cases by pathological examination, including 14 cases of submucosal leiomyoma and 9 cases of atypical polypoid adenomyosis. Additionally, we excluded three patients who underwent laparoscopic surgery for the repair of uterine perforation that occurred during hysteroscopic surgery. In total, 424 cases remained for analysis, and among them, we defined cases in which the target polyps were not detected by postoperative pathological examination as SRP. Specifically, these were cases in which only normal endometrium was detected in the surgical specimen. Multivariate analysis was performed to detect factors that were significantly related to SRP. For each patient, we recorded the number of polyps and additional clinical data, including the patient’s body mass index (BMI), parity, presence of hormonal drug use before surgery, and gynecological symptoms. Hormonal drugs included Sophia A (a tablet containing 1.0 mg norethisterone and 0.05 mg mestranol, ASKA Pharmaceutical Co., Ltd., Tokyo, Japan), Planovar (a tablet containing 0.5 mg norgestrel and 0.05 mg ethinyl estradiol, ASKA Pharmaceutical Co., Ltd., Tokyo, Japan), Dinagest (a tablet containing 1.0 mg dienogest, MOCHIDA Pharmaceutical Co., Ltd., Tokyo, Japan), and Relumina (a tablet containing 40 mg relugolix). Gynecological symptoms included infertility, abnormal bleeding, and hypermenorrhea. One patient reported multiple symptoms, and some patients had no complaints.

For the multivariate analysis, data on the following nine factors, other than the size of the target polyp, were extracted: “Advanced age” (≥ 42 years), “High BMI” (≥ 25 kg/m^2^), “Nulliparous,” “Single polyp,” “Infertility,” “Abnormal bleeding,” “Hypermenorrhea,” “No symptoms,” and “Hormonal drug use.” Since previous studies have demonstrated that polyps less than 10 mm in size were more likely to regress spontaneously within 1 year, “Small polyp” was defined as a polyp smaller than 10 mm in diameter [1, 7, 8, 10]. Since this study contained nearly 200 infertile patients, “Advanced age” was defined as patients aged 42 years or older with reference to some previous reports [11, 12].

### Detailed analysis of patients by classifying the size of the target polyp

To analyze the relationship between the size of the target polyp and the regression, we classified cases into the following five groups according to the maximal polyp size measured by transvaginal ultrasonography in an outpatient setting: ≤ 4.9 mm, 5.0–9.9 mm, 10.0–14.9 mm, 15.0–19.9 mm, and ≥ 20.0 mm. Additionally, to determine the most appropriate target polyp borderline size, a similar multivariate analysis of ten factors was performed with two other definitions of “small polyps” (that is, smaller than 15 mm or 5 mm, with no significant difference detected).

### Statistical analysis

All statistical analyses were performed using JMP version 12 for Windows (SAS Institute Inc., Tokyo, Japan). To eliminate confounding factors, we divided the patients into two groups according to the presence of each factor and performed a multivariate logistic regression analysis. For all patients, we assessed the influence of ten factors. The odds ratios (ORs) along with the 95% confidence intervals (CIs) were estimated to indicate the strength of these correlations. A *p* value < 0.05 was considered statistically significant. Other data are presented as the mean ± standard deviation.

## Results

### Relationship between frequency of SRP and polyp size

Patient characteristics are presented in Table 1, and the average patient age, BMI, target polyp size, and operation time were 39.2 ± 8.1 years, 21.6 ± 3.1 kg/m^2^, 13.3 ± 6.8 mm, and 16.3 ± 8.7 minutes, respectively. Among 424 cases, 368 were referred from another hospital for hysteroscopic polypectomy, and the average period between the first visit and operation was 54.8 ± 32.2 days; however, among other cases of patients who visited our hospital directly, the average period was 79.0 ± 40.8 days. In total, SRP was detected in 6.6% of all cases (*n* = 28/424, Table 2). When the frequencies of SRP were calculated in accordance with polyp size, the results were as follows: (1) 11.9% (*n* = 5/42) for “less than 4.9 mm”; 16.5% (*n* = 18/109) for “5.0–9.9 mm”; 2.8% (*n* = 4/145) for “10.0–14.9 mm”; 1.4% (*n* = 1/73) for “15.0–19.9 mm”; and 0.0% (*n* = 0/55) for “over 20.0 mm” (Table 2). From this result, polyps less than 10 mm could be considered the criterion for predicting a high likelihood of SRP.

**Table 1:** Patient characteristics

Characteristics	Avg. ± SD (Min–Max), Number
Age (years old)	39.2 ± 8.1 (22–86), *n* = 424
Parity	0.55 ± 0.86 (0–4), *n* = 424
BMI (kg/m^2^)	21.6 ± 3.1 (16.3–40.2), *n* = 424
Polyp size (mm)	13.4 ± 6.8 (3.0–55.2), *n* = 399
Operation time (minutes)	16.3 ± 8.7 (2–73), *n* = 424
Hormonal drug use	*n* = 258
Norethisterone–mestranol	*n* = 84
Norgestrel–ethinyl estradiol	*n* = 38
Dienogest	*n* = 114
Relugolix	*n* = 16
Others	*n* = 6
Symptoms	
Infertility	*n* = 174
Abnormal bleeding	*n* = 96
Hypermenorrhea	*n* = 81
No symptom	*n* = 98

Data on representative patient characteristics obtained from medical records are summarized in this table. For each item, we calculated the average and standard deviation, minimal and maximal value, and count data from medical records. “Polyp size” refers to the maximum length of the target polyp measured by transvaginal ultrasonography before hospitalization in an outpatient setting. In the calculation of polyp size, 25 cases described as having a “small polyp” without specific figures in the medical records were excluded.

*Avg.* average, *BMI* body mass index, *Min* minimum, *Max* maximum, *SD* standard deviation

**Table 2:** Relationship between polyp size and spontaneous regression rate

Polyp size	% (regression/total cases)
Total	6.6% (*n* = 28/424)
Less than 4.9 mm	11.9% (*n* = 5/42)
5.0–9.9 mm	16.5% (*n* = 18/109)
10.0–14.9 mm	2.8% (*n* = 4/145)
15.0–19.9 mm	1.4% (*n* = 1/73)
Over 20.0 mm	0.0% (*n* = 0/55)

“Polyp size” refers to the maximum length of the target polyp measured by transvaginal ultrasonography before hospitalization in an outpatient setting.

### Predictive factors of SRP

To detect factors that significantly affected the frequency of SRP, we compared the ten aforementioned factors using multivariate logistic regression models. The number of patients with each factor and the results of this analysis are presented in Table 3. In this analysis, “Small polyps” (OR 9.6, 95% CI 3.6–25.9, *p* < 0.01, Table 3) and “Hormonal drug use” (OR 4.2, 95% CI 1.4–12.2, *p* < 0.05, Table 3) were found to significantly impact the frequency of SRP, whereas the remaining eight factors, including representative symptoms of uterine endometrial polyps, did not show a significant impact. The patients who had the factors of “Small polyp” and “Hormonal drug use” had a respective SRP probability of 15.2% (*n* = 23/151) and 9.3% (*n* = 24/258). Additionally, the patients who had both factors had a SRP probability of 20.2% (*n* = 20/99).

**Table 3:** Identification of influencing factors for spontaneously regressed polyps

Factors	Number	OR (95% CI, number)	*p*-Value
Advanced age	144	0.5 (0.2–1.3, *n* = 6/28)	0.64
Nullipara	280	1.1 (0.5–2.5, *n* = 19/28)	0.49
High BMI	49	0.6 (0.1–2.5, *n* = 2/28)	0.80
Hormonal drug use	258	4.2 (1.4–12.2, *n* = 24/28)	0.02
Small polyp	151	9.6 (3.6–25.9, *n* = 23/28)	< 0.01
Single polyp	227	0.9 (0.4–1.8, *n* = 14/28)	0.72
Infertility	174	1.7 (0.8–3.7, *n* = 15/28)	0.66
Abnormal bleeding	96	0.7 (0.3–2, *n* = 5/28)	0.64
Hypermenorrhea	81	1.8 (0.8–4.2, *n* = 8/28)	0.20
No symptom	98	0.4 (0.1–1.3, *n* = 3/28)	0.96

A multivariate analysis of 424 patients with hysteroscopic polypectomy was performed to examine the influence of ten representative factors whose data were collected from medical records. The number of patients with each factor, the ORs and 95% CIs for the occurrence of spontaneously regressed polyps and the *p*-values are presented in this table. “Hormonal drug use” and “Small polyp” were identified as significant factors for the occurrence of spontaneous regression.

*BMI* body mass index, *CI* confidence interval, *OR* odds ratio

## Discussion

The management of endometrial polyps during infertility treatment has become increasingly important. In this study, infertility was also the most frequent indication for hysteroscopic polypectomy (41.0%, *n* = 174/424) since hysteroscopic polypectomy for infertile patients was effective, especially when the target polyp was large [6]. However, in other studies on the natural history of detected polyps, the spontaneous regression rate of endometrial polyps was documented after 1 year of observation [1, 7, 8]. The management of polyps is controversial owing to this phenomenon of SRP. Given the recovery period of the uterine endometrium after surgery [9] and the cost of surgery, surgical management should be avoided, when possible. Additionally, there is another problem: the 1-year observation period is too long for infertile patients. We also planned to perform hysteroscopic polypectomy promptly after diagnosis in most cases, and the observation period was limited to a shorter period as a result. The average time between the first visit and surgery was less than 2 months, namely, 58.0 ± 34.4 days. Even during this relatively short period and in cases in which small polyps were investigated strictly by pathological examination, SRP was detected in 28 cases, which made up 6.6% of the 424 total cases. This result indicated the possibility that some endometrial polyps were regressed within several menstrual cycles. The rate of SRP was inversely related to the size of the target polyp, although the cut-off value for “Small polyp” was not consistent with a previous similar report [13]. Then, to identify significant factors for predicting SRP, we retrospectively extracted data on ten factors that seemed to be related to endometrial polyps. As expected, the multivariate analysis of these factors revealed that “Small polyp,” defined as a polyp smaller than 10 mm, and “Hormonal drug use” had a significant impact on the probability of SRP. On the other hand, we could not detect a significant difference in any of the symptoms that were induced by endometrial polyps, including infertility, abnormal bleeding, and hypermenorrhea. This result supported the finding of a previous study, which concluded that hormonal drug treatment may have a role in the management of endometrial polyps if the target polyp is small [1, 14]. As the most important finding of this analysis was the impact of small polyp size and hormonal drug use, patients with these two factors might be offered treatment options other than surgery because in over 20% of these cases (20.2%, *n* = 20/99) endometrial polyps could not be detected by hysteroscopic polypectomy. In these cases, when the target polyp could not be clearly detected just before surgery, such as by transvaginal ultrasonography, cancellation of the operation might become a realistic option. However, there are some limitations to this study. First, in the present study, polyps smaller than 5 mm in diameter had a lower probability of regression than polyps measuring 5.0–9.9 mm in diameter. This may have been because these polyps were difficult to detect and diagnose or because the patients underwent conservative treatments. Second, we did not consider the form of the target polyp, such as whether it was sessile or pedunculated, even though previous studies have indicated that sessile polyps are more likely to regress following oral contraceptive treatment than pedunculated polyps [14]. In this study, we could not search for data on this polyp shape difference since we retrospectively collected data about examination and operation records. A larger study is needed to address these limitations.

## Conclusions

We found that endometrial polyps smaller than 10 mm might not require prompt surgical treatment, since the target polyps in more than 15% of these cases spontaneously regressed within approximately 2–3 months. Furthermore, we found that hormonal drugs should be used for conservative treatment. The patients who had both factors, namely, “Small polyp” and “Hormonal drug use,” could be expected to have a greater than 20% possibility of polyp regression. Conversely, if a polyp is larger than 10 mm, polyps cannot regress within the short term.

## Data Availability

The authors agree to make all data from this study freely available.

## Abbreviations

BMI: Body mass index
CI: Confidence interval
OR: Odds ratio
SRP: Spontaneously regressed polyp
